# Supplementary material for: Fueling ab initio folding with marine metagenomics enables structure and function predictions of new protein families
Source: Genome Biol. 2019 Nov 1;20:229. doi: 10.1186/s13059-019-1823-z (PMC6825341; doi:10.1186/s13059-019-1823-z)
Supplement: Supplementary file 1 — Additional file 1: Figure S1. Microbial community profiles at phylum and genus levels in 132 datasets. Figure S2. Scattering plot of Nf values for 2,801 Pfam families searched through the IMG+Uniref100 versus that searched through the Tara+UniRef100 datasets. Figure S3. A breakdown of the Pfam families based on different metagenome database searches. Figure S4. Taxonomical distribution of all the genera in the Pfam families that are modellable using different sequence samples. Figure S5. Species distribution for 797 Pfam families modeled with the combined Tara and MetaClust dataset. Figure S6. Summary of predicted models on 417 Pfam families by C-QUARK using a combined Tara Oceans and MetaClust metagenome dataset. Figure S7. Representative C-QUARK structure models predicted using a combined Tara Oceans and MetaClust sequence dataset. Table S1. A breakdown of the samples in the IMG database on Feb 21, 2017. Table S4. Detailed information of Gene distribution for Tara oceans dataset. Table S4. Summary of Nf score and TM-score for the 27 Pfam families modeled. Table S6. Structure-based function annotations on 27 Pfam families selected. Table S8. Comparison between the first models predicted by C-QUARK and PconsFold2 on a common set of 33 Pfam families. [file 13059_2019_1823_MOESM1_ESM.pdf]

Additional File 1

Table of Content

Supporting Figures

Fig. S1. Microbial community profiles at phylum and genus levels in 132 datasets.

Fig. S2. Scattering plot of Nf values for 2,801 Pfam families searched through the IMG+Uniref100 versus that searched through the Tara+UniRef100 datasets.

Fig. S3. A breakdown of the Pfam families based on different metagenome database searches.

Fig. S4. Taxonomical distribution of all the genera in the Pfam families that are modellable using different sequence samples.

Fig. S5. Species distribution for 797 Pfam families modeled with the combined Tara and MetaClust dataset.

Fig. S6. Summary of predicted models on 417 Pfam families by C-QUARK using a combined Tara Oceans and MetaClust metagenome dataset.

Fig. S7. Representative C-QUARK structure models predicted using a combined Tara Oceans and MetaClust sequence dataset.

Supporting Tables

Table S1: A breakdown of the samples in the IMG database on Feb 21, 2017.

Table S4: Summary of Nf score and TM-score for the 27 Pfam families modelled.

Table S6: Structure-based function annotations on 27 Pfam families selected.

Table S8: Comparison between the first models predicted by C-QUARK and PconsFold2 on a common set of 33 Pfam families.

Supplementary Figures

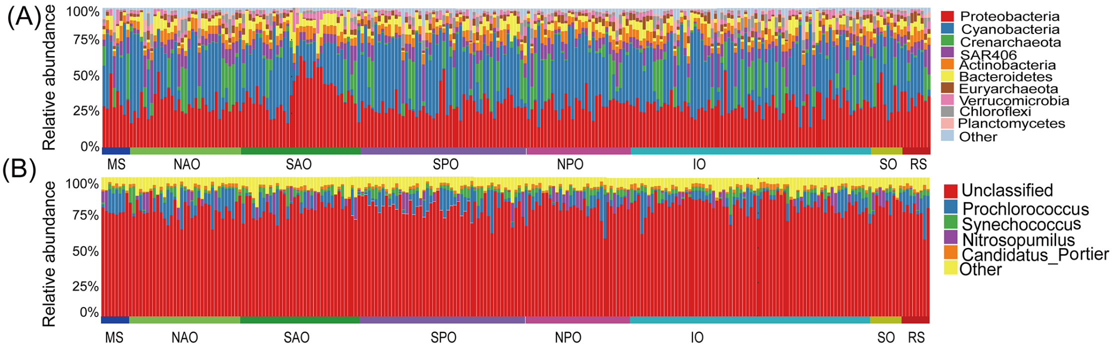

Fig. S1. Microbial community profiles at phylum and genus levels in 132 datasets. (A) Microbial community profiles of the top-ten phyla at phylum level. Vertical axis represents the relative abundance for each phylum. The 132 datasets are sorted by the ocean and sea regions. (B) Microbial community profiles of the top-five genera at genus level. Vertical axis represents the relative abundance for each genus. The 132 datasets are sorted by the ocean and sea regions and the regions are labeled beneath.

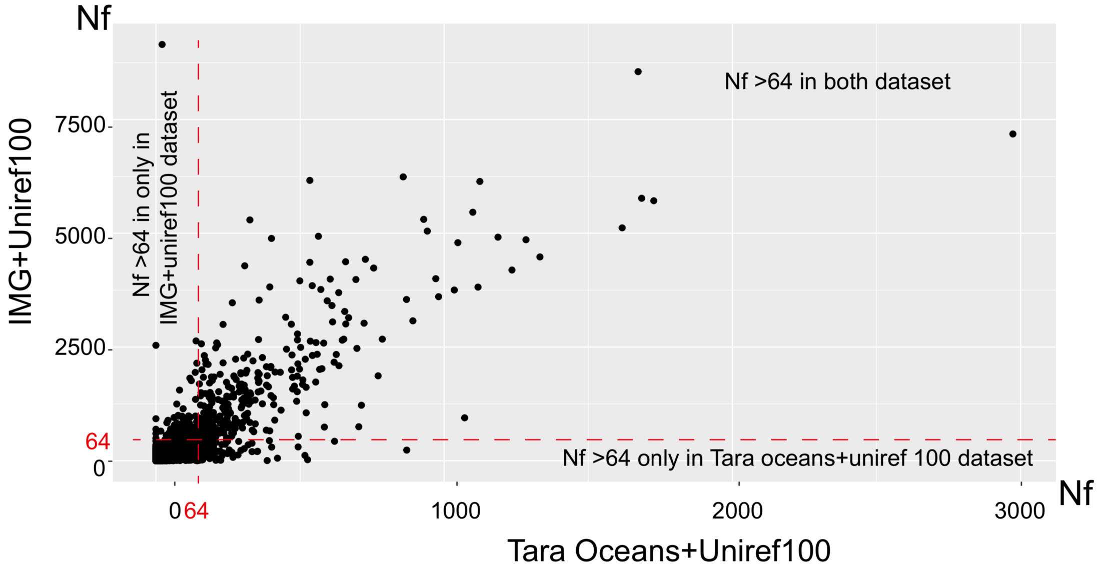

Fig. S2. Scattering plot of Nf values for 2,801 Pfam families searched through the IMG+Uniref100 versus that searched through the *Tara*+UniRef100 datasets.

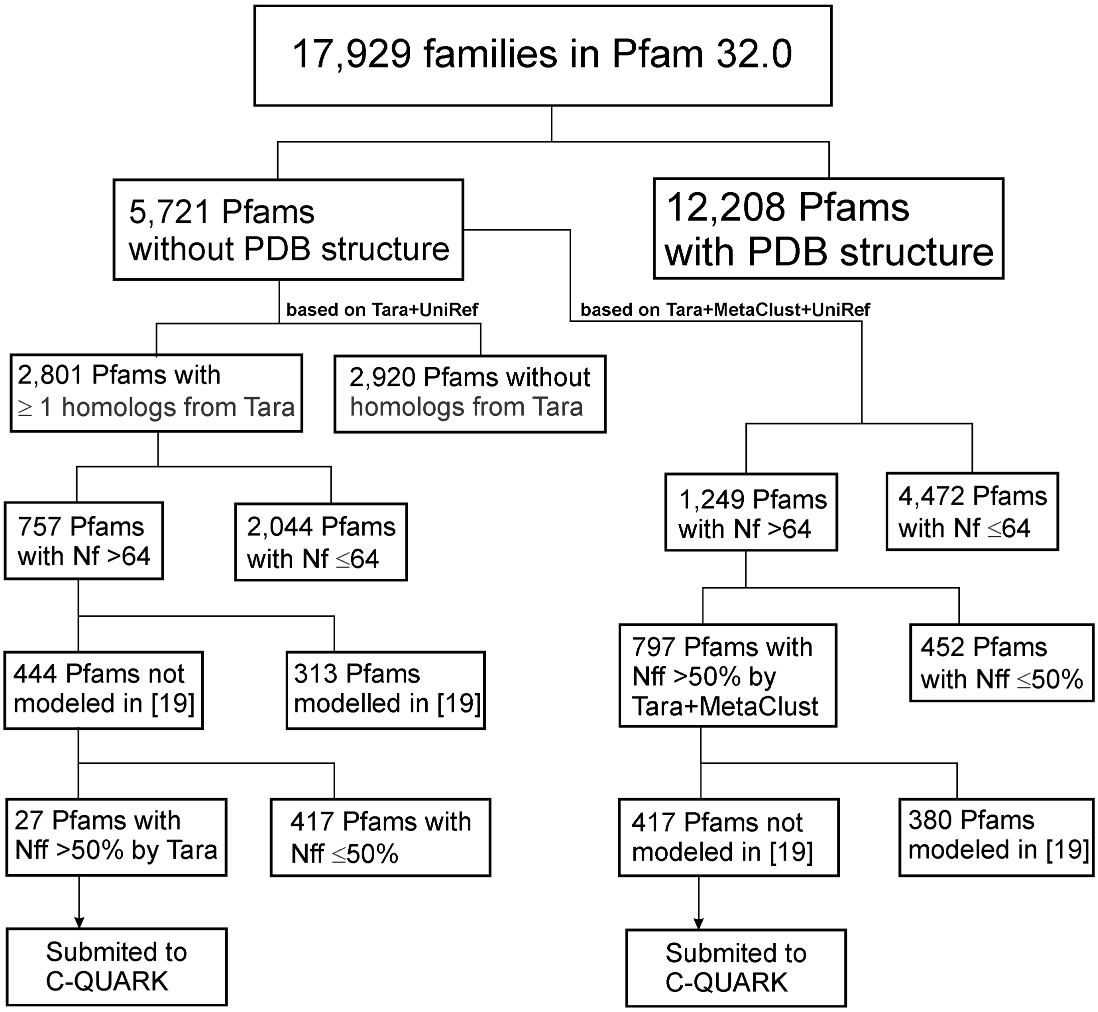

Fig. S3. A breakdown of the Pfam families based on different metagenome database searches.

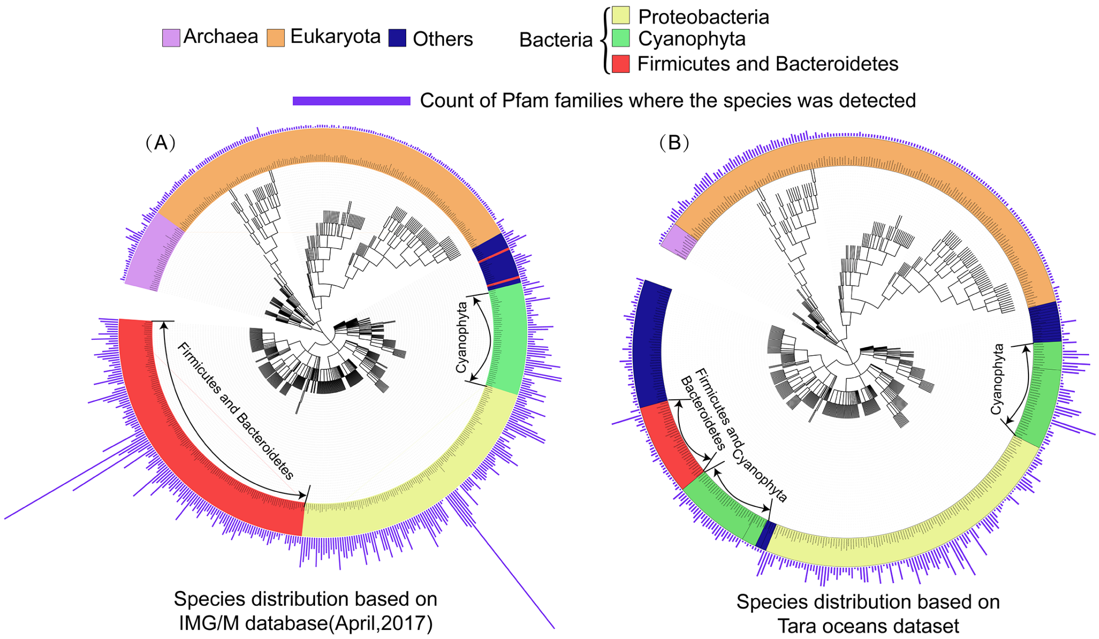

Fig. S4. Taxonomical distribution of all the genera in the Pfam families that are modellable using different sequence samples. Here, 'modellable' refers to the cases with Nf >64 using the IMG set by Ovchinnikov *et al.* or the case with Nf >64 and Nff >0.5 using *Tara* Oceans set in this study. (A) using IMG database with 614 Pfam families; (B) using *Tara* Oceans with 27 Pfam families. Different colors represent different phylum, and the bar corresponding to the outer circle represents the count of the Pfam families where the species was detected. Phylum *Bacteroidetes* and *Firmicutes* (labeled red in two panels), which are common in the gut microbiome according to taxonomical database and literature review, account for the overwhelming majority in the Pfam families modellable using the IMG. The phylum *Cyanophyta* (labeled green in two panels), which is dominant in the ocean microbiome, are more prevalent in the Pfam families modellable using the *Tara* Oceans dataset.

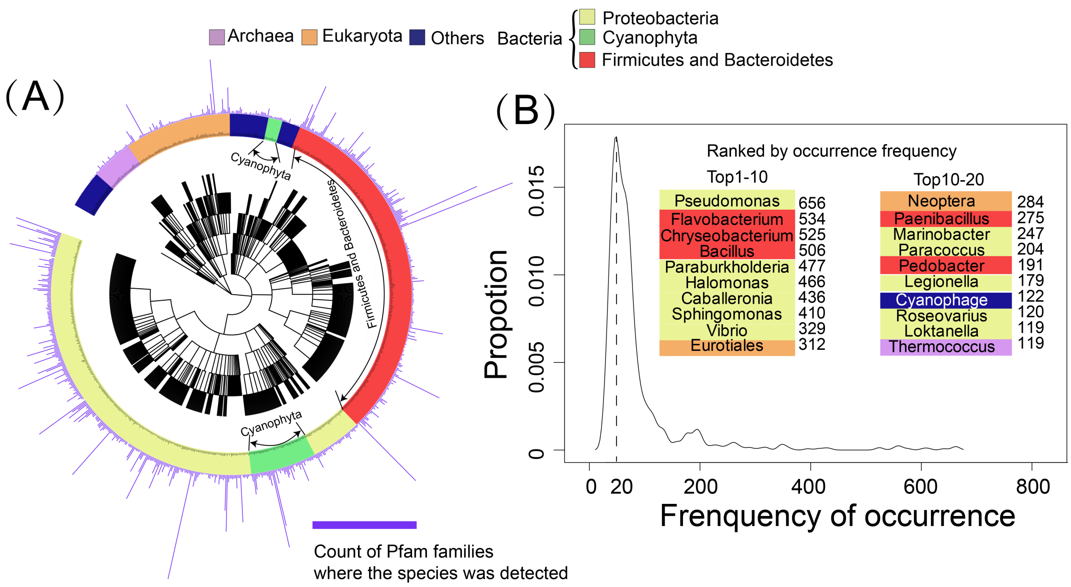

Fig. S5. Species distribution for 797 Pfam families modeled with the combined *Tara* and MetaClust dataset. (A) Species distribution for 797 Pfam families based on the record in Pfam database. Different colors represent different phylum, and the bar corresponding to the outer circle represents the count of Pfam families where the species was detected. (B) Histogram of occurrence frequency of species in the 797 Pfam families. The vertical axis represents the percentage of species with a specific frequency. Top 20 records ranked by occurrence frequency are illustrated and labeled with different colors corresponding to their phylum.

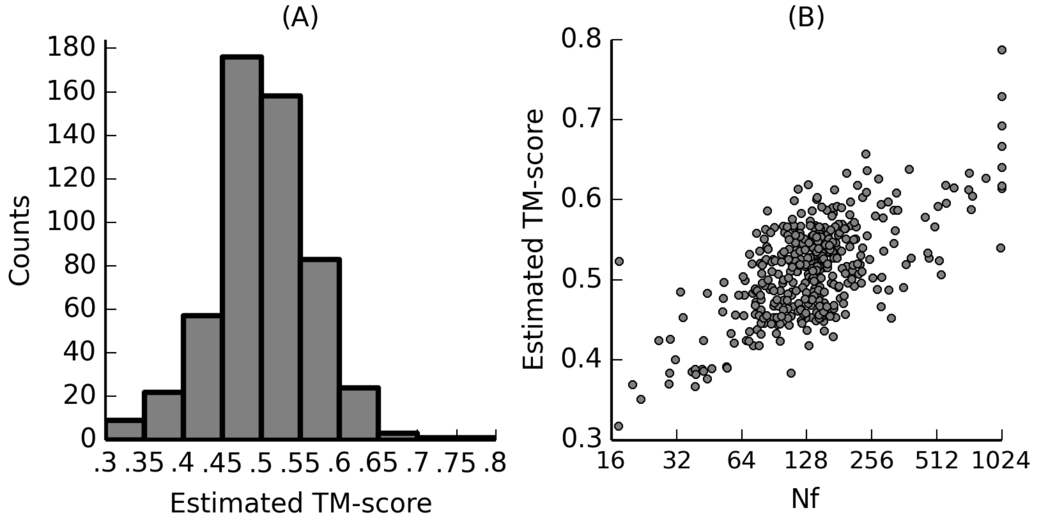

Fig. S6. Summary of predicted models on 417 Pfam families by C-QUARK using a combined *Tara* Oceans and MetaClust metagenome dataset. (A) Histogram of estimated TM-score. (B) Estimated TM-score versus Nf (plotted in log scale) for each target.

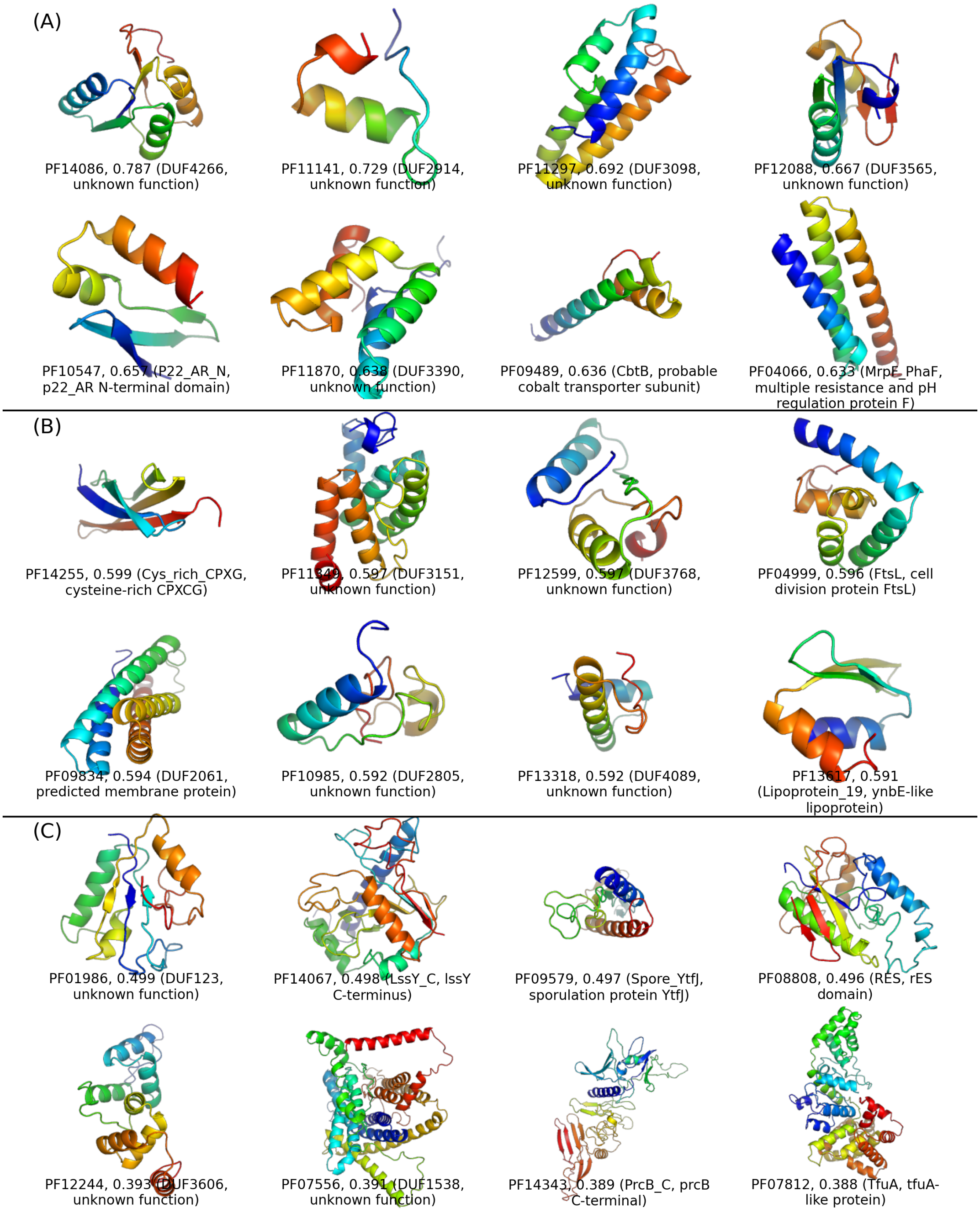

Fig. S7. Representative C-QUARK structure models predicted using a combined *Tara* Oceans and MetaClust sequence dataset. 416 predicted structures are ranked in descending order of estimated TM-scores, which are further divided into three bins: (A) [0.6, 1], (B) [0.5, 0.6), and (C) [0,0.5). Within each bin, eight examples are randomly selected as shown.

Supplementary Tables

Table S1: A breakdown of the samples in the IMG database on Feb 21, 2017. More than half of the samples (52.57%) were collected from gut microbiome of Human and Animal, while only 18.86% samples are from aquatic biomes. The value in the parentheses correspond to the data from the newest version of IMG on June 5, 2019, in which the number of samples was significantly increased in almost all the species and the percentages for gut microbiome of Human and Animal and aquatic biomes became 37.18% and 35.96% respectively.

Engineered	# Sample	Environment	# Sample	Host-associated	# Sample
Bioreactor	57 (186)	Air	50 (128)	Algae	18 (83)
Bioremediation	71 (96)	Aquatic	3217 (12115)	Animal	2012 (35)
Biotransformation	11 (17)	Terrestrial	3232 (4358)	Annelida	139 (146)
Built environment	100 (1259)			Arthropoda	112 (165)
Food production	0 (5)			Birds	17 (18)
Lab enrichment	164 (425)			Cnidaria	0 (11)
Lab synthesis	6 (7)			Fish	0 (4)
Modeled	23 (52)			Fungi	102 (113)
Solid waste	43 (77)			Human	6954 (12490)
Unclassified	6 (18)			Insecta	34 (34)
Wastewater	245 (544)			Invertebrates	7 (9)
				Mammals	196 (312)
				Microbial	10 (21)
				Mollusca	11 (12)
				Plants	200 (923)
				Porifera	12 (15)
				Tunicates	5 (9)

Table S4: Summary of Nf score and TM-score for the 27 Pfam families modelled. TM-score was estimated based on Eqs. (2-3) and data in Fig 6.

Pfam ID	NF-score	TM-score
PF02326	129.719	0.468
PF04380	193.668	0.699
PF05939	106.499	0.600
PF06067	126.857	0.504
PF06698	121.381	0.827
PF07583	298.922	0.534
PF07586	103.093	0.507
PF07587	350.121	0.558
PF07624	375.896	0.783
PF07626	161.627	0.533
PF07627	156.347	0.628
PF07631	210.411	0.635
PF07637	301.358	0.365
PF07864	86.426	0.639
PF08855	72.623	0.711
PF09834	231.879	0.560
PF09923	97.131	0.525
PF10985	84.408	0.655
PF11233	69.306	0.422
PF11297	98.810	0.623
PF11351	76.644	0.421
PF11360	81.847	0.516
PF11753	279.646	0.320
PF12322	173.175	0.579
PF14108	138.911	0.429
PF15461	105.133	0.414
PF16316	104.687	0.549

Table S6: Structure-based function annotations on 27 Pfam families selected. Gene Ontology is predicted by MetaGO program with the conference measured by Fscore. Gene Ontology is described in three aspects: Cellular Component, Biological Process and Molecular Function. 'NA' means that the function could not be defined.

Pfam ID	Go ID	Fscore	Function
Biological Process			
PF07586	GO:0055114	0.01	oxidation-reduction process
PF07627	GO:0071897	0.01	DNA biosynthetic process
PF16316	GO:0048013	0.01	ephrin receptor signaling pathway
PF07637	GO:0055114	0.01	oxidation-reduction process
PF07864	GO:2000679	0.01	positive regulation of transcription regulatory region DNA binding
PF12322	GO:0006567	0.01	threonine catabolic process
PF16316	GO:0048013	0.01	ephrin receptor signaling pathway
PF07587	GO:0044763	0.02	single-organism cellular process
PF11351	GO:0050658	0.02	RNA transport
PF11753	GO:0071704	0.02	organic substance metabolic process
PF11360	GO:0018199	0.02	peptidyl-glutamine modification
PF06067	GO:0044710	0.03	single-organism metabolic process
PF07583	GO:0046034	0.03	ATP metabolic process
PF07624	GO:0019682	0.03	glyceraldehyde-3-phosphate metabolic process
PF09834	GO:0051234	0.03	establishment of localization
PF10985	GO:2000112	0.03	regulation of cellular macromolecule biosynthetic process
PF11233	GO:0044249	0.03	cellular biosynthetic process
PF07626	GO:0007017	0.04	microtubule-based process
PF07631	GO:0044710	0.04	single-organism metabolic process
PF11246	GO:0065008	0.04	regulation of biological quality
PF09923	GO:0009987	0.35	cellular process
PF15461	GO:0044710	0.53	single-organism metabolic process
PF15461	GO:0044710	0.53	single-organism metabolic process
PF08855	GO:0009987	0.87	cellular process
PF04380	GO:0009987	0.98	cellular process
PF06698	GO:0071704	1	organic substance metabolic process
Molecular Function			
PF11246	GO:0052689	0.05	carboxylic ester hydrolase activity
PF04380	GO:0003824	0.92	catalytic activity
PF08855	GO:0003824	0.22	catalytic activity
PF09923	GO:0003824	0.22	catalytic activity
PF06067	GO:0043169	0.03	cation binding
PF07624	GO:0043169	0.04	cation binding
PF10985	GO:0043169	0.02	cation binding
PF11360	GO:0043169	0.04	cation binding
PF07631	GO:0050660	0.05	flavin adenine dinucleotide binding
PF07583	GO:0020037	0.02	heme binding
PF11233	GO:0016810	0.03	hydrolase activity, acting on carbon-nitrogen (but not peptide) bonds
PF11753	GO:0016798	0.05	hydrolase activity, acting on glycosyl bonds
PF07864	GO:0003950	0.01	NAD+ ADP-ribosyltransferase activity
PF15461	GO:0016491	0.59	oxidoreductase activity
PF15461	GO:0016491	0.59	oxidoreductase activity
PF07587	GO:0035639	0.03	purine ribonucleoside triphosphate binding
PF07626	GO:0035639	0.05	purine ribonucleoside triphosphate binding
PF06698	GO:0003735	1	structural constituent of ribosome
PF16316	GO:0016746	0.02	transferase activity, transferring acyl groups
PF16316	GO:0016746	0.02	transferase activity, transferring acyl groups
PF07627	GO:0016772	0.01	transferase activity, transferring phosphorus-containing groups
PF07586	NA		
PF11351	NA		

PF07637	NA		
PF09834	NA		
PF12322	NA		
Cellular Component			
PF04380	GO:0044464	1	cell part
PF09923	GO:0044464	0.97	cell part
PF11753	GO:0009986	0.01	cell surface
PF06698	GO:0044444	1	cytoplasmic part
PF14108	GO:0044444	1	cytoplasmic part
PF08855	GO:0044444	0.96	cytoplasmic part
PF07626	GO:0005856	0.06	cytoskeleton
PF10985	GO:0005829	0.01	cytosol
PF07864	GO:0070062	0.01	extracellular exosome
PF11360	GO:0005576	0.02	extracellular region
PF16316	GO:0032580	0.01	Golgi cisterna membrane
PF09834	GO:0016021	0.01	integral component of membrane
PF07587	GO:0005874	0.02	microtubule
PF11351	GO:0044611	0.01	nuclear pore inner ring
PF06067	GO:0030288	0.02	outer membrane-bounded periplasmic space
PF07627	GO:0042597	0.01	periplasmic space
PF11233	GO:0042597	0.01	periplasmic space
PF07583	GO:0070469	0.03	respiratory chain
PF15461	GO:0070469	0.02	respiratory chain
PF15461	GO:0070469	0.02	respiratory chain
PF16316	GO:0016746	0.02	transferase activity, transferring acyl groups
PF11246	GO:0098025	0.04	virus tail, baseplate
PF07586	NA		
PF07637	NA		
PF12322	NA		
PF07624	NA		
PF07631	NA		

Table S8: Comparison between the first models predicted by C-QUARK and PconsFold2 on a common set of 33 Pfam families. Targets are listed in descending order of TM-score between the PconsFold2 and C-QUARK models. Model and contacts of PconsFolds are downloaded from http://c3.pcons.net/static/download/PconsFold2_union.tar.gz.

Pfam ID	TM-score (C-QUARK & PconsFold2)[(a)]	Estimated TM-score of C-QUARK[(b)]	Contact satisfaction rate[(c)]	
			PconsFold2	C-QUARK
PF05670	0.610	0.568	0.312	1.000
PF11248	0.548	0.533	0.583	0.583
PF09523	0.508	0.558	0.350	0.800
PF04468	0.471	0.556	0.353	0.833
PF07040	0.460	0.561	0.206	0.878
PF00379	0.439	0.542	0.091	0.667
PF08310	0.428	0.569	0.200	0.200
PF10048	0.419	0.475	0.182	0.759
PF12276	0.406	0.486	0.467	0.788
PF13342	0.401	0.535	0.364	0.500
PF10882	0.376	0.369	0.150	0.345
PF14086	0.375	0.787	0.500	1.000
PF10601	0.365	0.446	0.000	0.444
PF14343	0.359	0.389	0.273	0.474
PF12625	0.359	0.510	0.158	0.262
PF13280	0.355	0.522	0.343	0.043
PF14041	0.350	0.279	0.353	0.266
PF16199	0.341	0.520	0.529	0.778
PF03504	0.326	0.527	0.222	0.441
PF14375	0.322	0.522	0.400	0.769
PF04463	0.320	0.492	0.385	0.038
PF01548	0.317	0.486	0.400	0.053
PF13116	0.316	0.485	0.224	0.600
PF14020	0.304	0.537	0.000	0.700
PF04448	0.288	0.550	0.231	0.462
PF01098	0.288	0.499	0.042	0.014
PF10517	0.288	0.523	0.400	0.400
PF14105	0.284	0.519	0.636	0.412
PF14255	0.265	0.599	0.200	1.000
PF12810	0.258	0.502	0.300	0.057
PF06995	0.242	0.487	0.625	0.080
PF05594	0.225	0.526	0.000	0.167
PF13634	0.216	0.551	0.222	0.375
**Average**	**0.348**	**0.500**	**0.285**	**0.476**

(a) TM-score between the first PconsFold2 and C-QUARK models, which is calculated by TM-align since the sequences selected by the two programs can be different. For a pair of two structures, TM-align outputs two TM-scores normalized separately by the length of each of the two proteins, where the larger TM-score is reported here.

(b) Estimated TM-score of the C-QUARK models.

(c) The portion of top $L/5$ long-range contacts that are satisfied in the predicted models with Cβ atom distances <8Å (or Cα atoms for glycines). Here, the original contact-maps from each pipeline are used to calculate the contact satisfaction rate.
